# Ultrahigh Oxidation Resistance and High Electrical Conductivity in Copper-Silver Powder

**DOI:** 10.1038/srep39650

**Published:** 2016-12-22

**Authors:** Jiaxiang Li, Yunping Li, Zhongchang Wang, Huakang Bian, Yuhang Hou, Fenglin Wang, Guofu Xu, Bin Liu, Yong Liu

**Affiliations:** 1 School of Materials Science and Engineering, Central South University, Changsha, China; 2 State Key Lab for Powder Metallurgy, Central South University, Changsha, China; 3 Advanced Institute for Materials Research, Tohoku University, 2-1-1 Katahira, Aoba-ku, Sendai 980-8577, Japan; 4 Institute for Materials Research, Tohoku University, 2-1-1 Katahira, Sendai, Miyagi 980-8577, Japan

## Abstract

The electrical conductivity of pure Cu powder is typically deteriorated at elevated temperatures due to the oxidation by forming non-conducting oxides on surface, while enhancing oxidation resistance via alloying is often accompanied by a drastic decline of electrical conductivity. Obtaining Cu powder with both a high electrical conductivity and a high oxidation resistance represents one of the key challenges in developing next-generation electrical transferring powder. Here, we fabricate a Cu-Ag powder with a continuous Ag network along grain boundaries of Cu particles and demonstrate that this new structure can inhibit the preferential oxidation in grain boundaries at elevated temperatures. As a result, the Cu-Ag powder displays considerably high electrical conductivity and high oxidation resistance up to approximately 300 °C, which are markedly higher than that of pure Cu powder. This study paves a new pathway for developing novel Cu powders with much enhanced electrical conductivity and oxidation resistance in service.

For electrical transferring powders applied in numerous areas such as electronic packaging, printed electronic devices and electromagnetic interference shielding^1^,^2^,^3^,^4^,^5^, high electrical conductivity and good oxidation resistance together are often required. Despite that the pure metal powders such as Cu show high conductivity, they usually demonstrate extremely low oxidation resistance. After oxidation, the rapid coverage of non-conductive oxide scales on surface results in an insulating effect between Cu particles, which in turn deteriorates significantly electrical conductivity of Cu powder. On the other hand, Ag powders possess high oxidation resistance and high electrical conductivity, but they are expensive and exhibit the electro-migration phenomena at elevated temperatures, which obstruct their widespread applications^6^,^7^.

Enhancing oxidation resistance whilst maintaining high electrical conductivity of Cu powder has been a subject of intensive research in the last decades because of both the fundamental scientific importance and the technological applications. However, improving oxidation resistance of Cu powder via alloying is generally accompanied by a dramatic decrease in electrical conductivity. For example, alloying Cu with Ti, Mg, Al, Pd, Ag, Nb or Cr can raise oxidation resistance of Cu by several orders of magnitude^8^,^9^, but the electrical conductivity falls to 10–30% of pure Cu^10^,^11^. This is attributed to the fact that the alloying elements tend to increase the scattering rate of conducting electrons at point defects, leading to the increase in electrical resistivity of the metal^12^. Alternatively, coating Cu particle with Ag by chemical processes seems to represent a prospective way of promoting oxidation resistance without sacrificing the electrical conductivity^13^,^14^,^15^. However, such enhancement turns out not to be striking due to the low adhesive force at the Cu-core/Ag-shell interface and also for the loose microstructures of Ag shells on Cu particles^16^,^17^ that are unable to obstruct the inward diffusion of O. For electrical transferring Cu powder, these two essential properties, high electrical conductivity and high oxidation resistance, seems always contradictory.

Oxidation of metals is sensitive to chemical composition and surface structure, and often occurs first from the crystalline defects such as grain boundaries (GBs) and dislocation sites owing to their high Gibbs free energy, and then spreads into the entire surface^18^. Therefore, the key to producing conducting yet high oxidation-resistant Cu powder is to seek an appropriate microstructure in which the initiation of oxidation at crystalline defects is effectively retarded while the scattering effect of conducting electrons in particles is minimized. It is well acknowledged that GBs can often serve as preferred sites for secondary phase precipitation because of their high interfacial energy possibly originated from the disordered arrangement of atoms and the higher proportion of lattice defects compared to matrix^19^. It is hence considered that only if the precipitates on these defects are chemically inert yet electrically conducting, the initiation of oxidation could be inhibited greatly. In this regard, if the particles are characterized by a high density of conducting and inert precipitates at GBs while the matrix is free of alloying elements, metal powders are expected to exhibit ultrahigh oxidation resistance without sacrificing electrical conductivity.

Cu-Ag alloy powder may be an ideal candidate to fulfill this hypothesis because Ag and Cu are utterly inter-dissolvable at high temperature, while at low temperature, most of Ag precipitates out from Cu preferentially along GBs^20^. This will give rise to Cu particles featured by Ag network along the GBs, consequently purifying Cu matrix. Such Ag network along GBs is helpful to enhance the oxidation resistance via hindering preferential oxidation due to the intrinsic inert nature of Ag. On the other hand, due to the close lattice parameters of Ag and Cu, residual Ag in Cu matrix has been proven not to be detrimental to the electrical conductivity of Cu^21^,^22^,^23^. Here, we fabricate such Cu-Ag powder with continuous Ag network distributed along GB of Cu particles and demonstrate that this new structure can inhibit the preferential oxidation at GB even at elevated temperature. As a result, Cu-Ag powder displays considerably high electrical conductivity and high oxidation resistance up to approximately 300 °C, markedly higher than pure Cu powder.

The composition of Cu-Ag alloy was determined by using Cu-Ag binary phase diagram^24^. For Cu-5 wt%Ag alloy, Cu and Ag are completely soluble to each other at temperature higher than 600 °C, and at lower temperature Ag will precipitate from Cu matrix preferentially along the lattice defects such as BGs. Alloy with higher Ag concentration would give rise to much rougher surface after precipitation, which is harmful to the flowablity of powder from our preliminary research.

## Results

**Figure 1:**
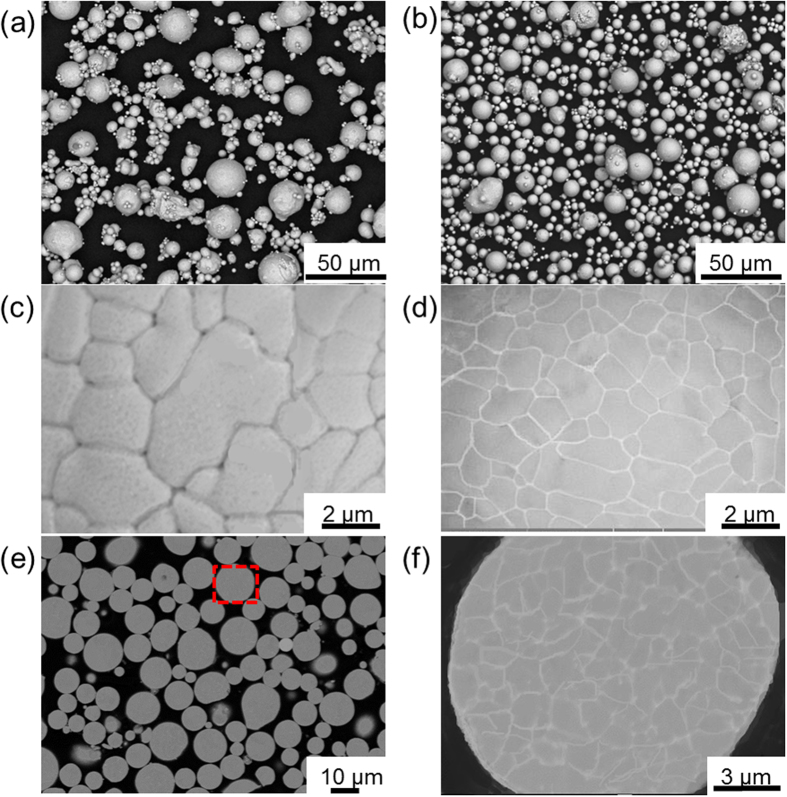
Microstructure of three Cu powders. (**a**,**b**) BEI images of the as-atomized pure Cu powder (**a**) and Cu-5Ag powder (**b**). (**c**,**d**) Enlarged BEI images highlighting the surface structure of the as-atomized Cu-Ag particle before (**c**)and after (**d**) aging at 250 °C for 60 min. (**e**,**f**) Cross-section view of the aged Cu-Ag powder (**e**) and the corresponding enlarged image (**f**).

**Figure 2:**
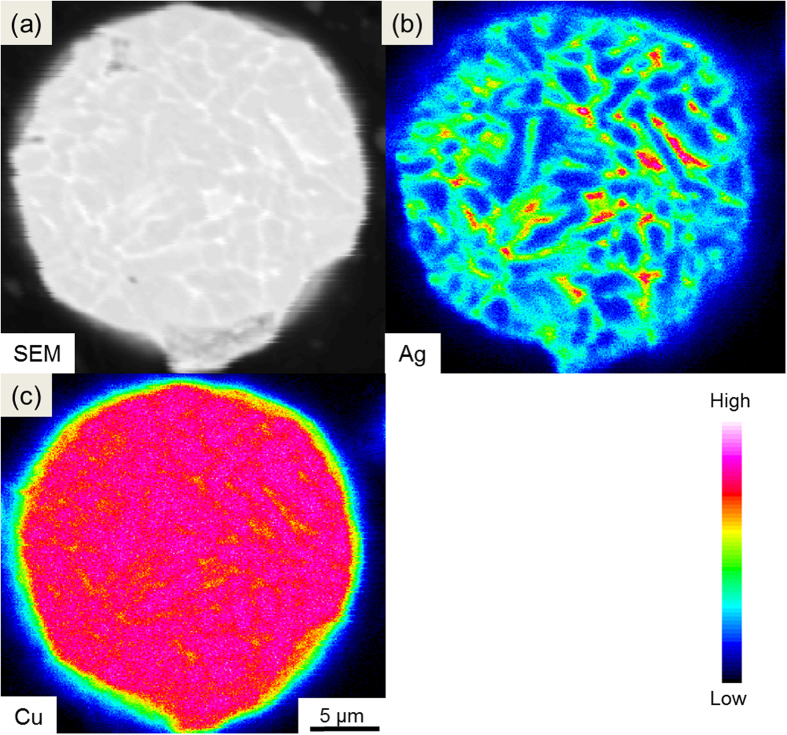
EPMA analysis. EPMA analysis of elemental distribution of Cu and Ag on the cross section of the Cu-Ag particle after aging at 250 °C for 60 min: (**a**) BEI image, (**b**) Ag, and (**c**) Cu.

Figure 1a,b shows the profiles of pure Cu and as-atomized Cu-Ag powders, both of which indicate that the microstructure is characterized by spherical particles with smooth surface. The GBs of the as-atomized Cu-Ag particles are visible and exhibit darker contrast in the BEI mode. After the aging treatment (250 °C, 1 h), the contrast of GBs turns bright, which suggests preferential precipitation of Ag along GBs (Fig. 1d), forming a continuous network. The cross-section view of the aging-treated Cu-Ag particles reveals that Ag atoms precipitate at GBs of the surface and the interior of particles, constituting a three-dimensional Ag network (Fig. 1e,f). Such distribution of Ag in the cross section of Cu particles can be observed more clearly by using EPMA elemental distribution (Fig. 2), in consistent with the Cu-Ag binary phase diagram. Although Cu and Ag are inter-dissolvable at high temperature, the solubility of Ag in Cu matrix decreases dramatically at lower temperature (250 °C)^25^,^26^, giving rise to the formation of continuous Ag network along GBs.

**Figure 3:**
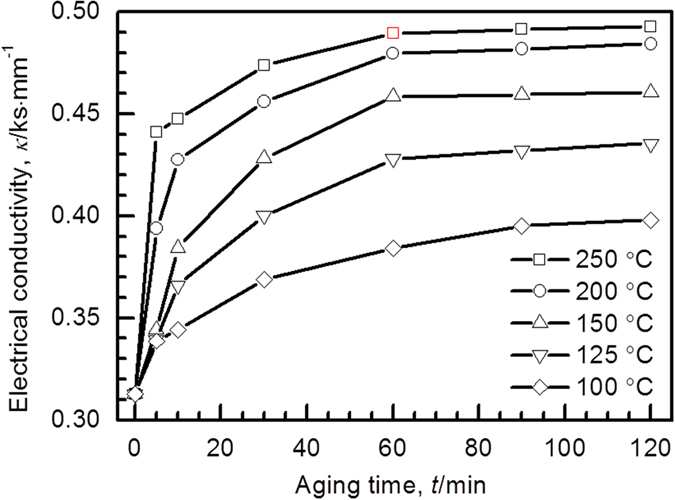
Aging time dependence of electrical conductivity. Electrical conductivity for Cu-Ag powder as a function of aging time up to 120 min. The aging temperature increases from 100 to 250 °C at an interval of 50 °C.

Figure 3 shows the electrical conductivity of Cu-Ag powder after aging at 100 to 250 °C. At each aging temperature, the electrical conductivity increases substantially before 40 min and then gradually reaches a constant value. For example, the electrical conductivity for the powder aged at 250 °C for 60 min reaches a maximum value of about 0.49 ks·mm^−1^, which is more than 50% higher than that of initial powder (0.31 ks·mm^−1^). For a given aging time, the electrical conductivity grows significantly with the aging temperature, while such tendency is not so obvious at the temperature above 200 °C implying that further rise in aging temperature is not beneficial to the electrical conductivity.

**Figure 4:**
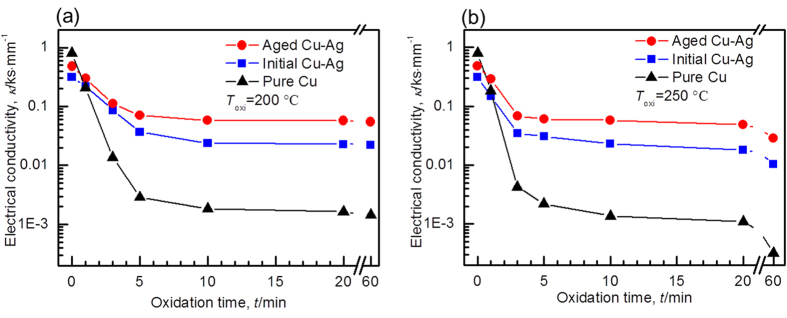
Electrical conductivity as a function of oxidation time. The change in the electrical conductivity during the oxidation process for the three types of Cu-based powders at (**a**) 200 °C and (**b**) 250 °C.

Figure 4a,b shows the electrical conductivity as a function of oxidation time for the Cu, as-atomized Cu-Ag and aging-treated Cu-Ag (250 °C, 1 h) powders. Although Cu powder displays the highest electrical conductivity in the beginning, its electrical conductivity falls sharply during oxidation in comparison to the other two powders. Note that at the two temperatures (200 °C and 250 °C), the aging-treated powder shows an extremely high electrical conductivity for a given exposure time, and its decreasing rate is the lowest among the three powders. For example, Cu powder at oxidation time of 0 min is as high as 0.78 ks·mm^−1^, which is nearly 1.5 times and 2.5 times higher than that of the aging-treated and as-atomized Cu-Ag powders. However, once after oxidation at 200 °C for 5 min, the electrical conductivity of Cu powder drops to as low as 2 × 10^−3^ ks·mm^−1^, which is 1/40 and 1/20 of that of the aging-treated and as-atomized Cu-Ag powders subjected to the comparable extent of oxidation, respectively. These results indicate that the Cu-Ag powder after aging exhibit both high electrical conductivity and high oxidation resistance, which can be attributed to the formation of a continuous Ag network along GBs.

**Figure 5:**
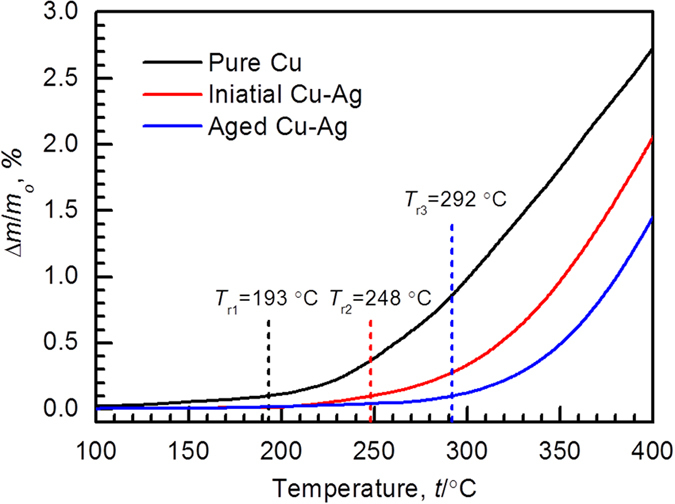
ThermogravimetricAnalysis. Thermogravimetric curves of three Cu-based alloy powders. The heating rate is 30 °C/min. The starting points for the oxidation, denoted as *T*_r1_, *T*_r2_ and *T*_r3_, are assumed upon the specific mass gain (*D*_m/mo_) hitting 0.1%.

Figure 5 shows mass change (Δ*m*/*m*) as a function of temperature obtained in flowing air at a heating rate of 30 °C/min, which the difference of oxidation behavior between the three powders can be seen clearly. The mass change of Cu powder varies insignificantly before 200 °C, yet goes up markedly with the increase of temperature. The same trend is observed for the as-atomized and aging-treated Cu-Ag powders, but their increase onset is higher for the aging-treated Cu-Ag than the as-atomized Cu-Ag. For a quantitative evaluation on the oxidation behavior, we choose the value of Δ*m*/*m* of 0.1% as the starting point of oxidation. The starting points of oxidation of pure Cu, as-atomized Cu-Ag, and aging-treated Cu-Ag powders are estimated to be 193, 248 and 292 °C, respectively, suggesting that the aging-treated Cu-Ag powder exhibits a much enhanced oxidation resistance in comparison to the as-atomized Cu-Ag and Cu powder by approximately 45 and 100 °C, respectively.

**Figure 6:**
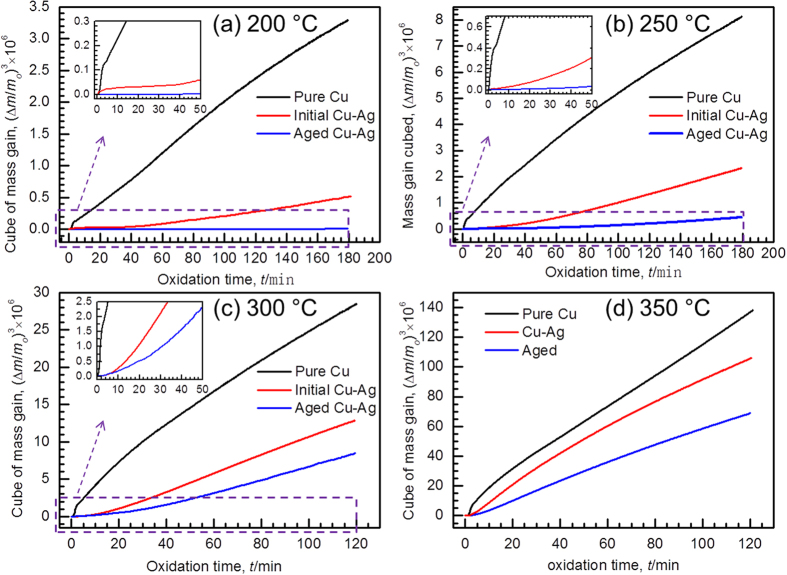
Oxidation isotherm. Oxidation isotherms for the three Cu-based powders at (**a**) 200 °C, (**b**) 250 °C, (**c**) 300 °C, and (**d**) 350 °C.

Figure 6 shows the mass change as a function of time for the three powders after oxidation at 200, 250, 300, and 350 °C. The oxidation rate for the two Cu-Ag powder samples is extraordinarily low in the beginning, while the mass change of Cu powder ascends much more substantially even at the very beginning (see the inserts in Fig. 6a–c). In contrast to the other two powders, the aging-treated Cu-Ag powder shows the highest oxidation resistance despite of the oxidation condition.

**Figure 7:**
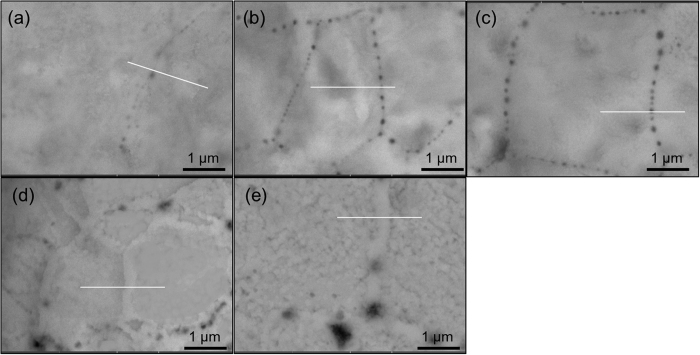
Structural evolution of the pure Cu. (**a**–**e**) Backscattered scanning electron images for the pure Cu powder after an oxidation period of 0 min (**a**), 1 min (**b**), 3 min (**c**), 5 min (**d**) and 10 min (**e**) at 200 °C.

**Figure 8:**
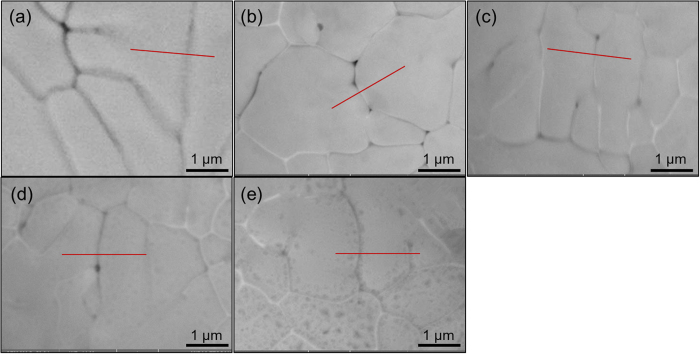
Structural evolution of the initial Cu-Ag powder. (**a**–**e**) Backscattered scanning electron images for theinitial Cu-Ag powder after an oxidation period of 0 min (**a**), 1 min (**b**), 3 min (**c**), 5 min (**d**) and 10 min (**e**) at 200 °C.

**Figure 9:**
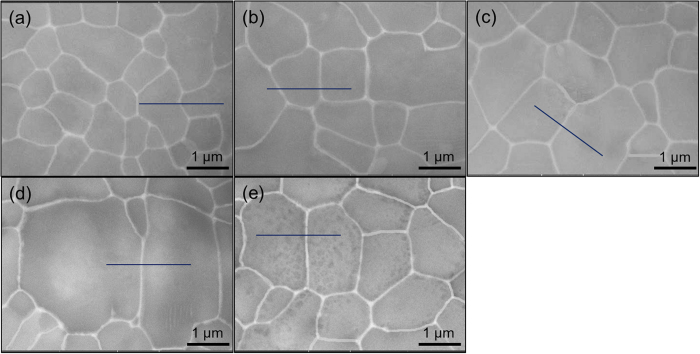
Structural evolution of the aged Cu-Ag powder. (**a**–**e**) Backscattered scanning electron images for the aged Cu-Ag powder after an oxidation period of 0 min (**a**), 1 min (**b**), 3 min (**c**), 5 min (**d**) and 10 min (**e**) at 200 °C.

Figures 7, 8 and 9 show the typical BEI images for particle surface of the three powders after oxidation at 200 °C for different durations. In the case of Cu powder, the GBs, which are characterized by black dots (oxide), are observed to be readily affected by O even under oxidation for 0 min (Fig. 7a). The dots become larger and the number of dots increases significantly with the oxidation proceeding (b to c). After oxidation for longer than 5 min, GBs become ambiguous and the interior of grain matrix becomes rough, which is characterized by larger black dots inside the grains. This suggests that oxidation of pure Cu occurs preferentially at GBs and extends into matrix thereafter.

In the case of the as-atomized Cu-Ag powder, since no aging treatment is carried out prior to oxidation, GBs are free of Ag precipitates before oxidation. In the oxidation process, the contrast of some GBs turns bright, suggestive of precipitation of Ag on GBs during oxidation. There are no dark dots observed at GBs with Ag precipitates, implying its inhibiting effect on oxidation surrounding GBs. Conversely, some GBs are free of Ag and the area along these GBs is full of dark dots. This again implies preferential oxidation along Ag-free GBs. Further microstructure observation of sample surface of the aging-treated Cu-Ag powder after various oxidation periods reveals an extraordinary oxidation resistance, as shown in Fig. 9. Invariably, GBs are characterized by continuous uniform distribution of Ag (white contrast) throughout the oxidation. Even in the case of oxidization after 10 min, the Ag network is clearly apparent, although there are a few of dark dots inside the grains.

**Figure 10:**
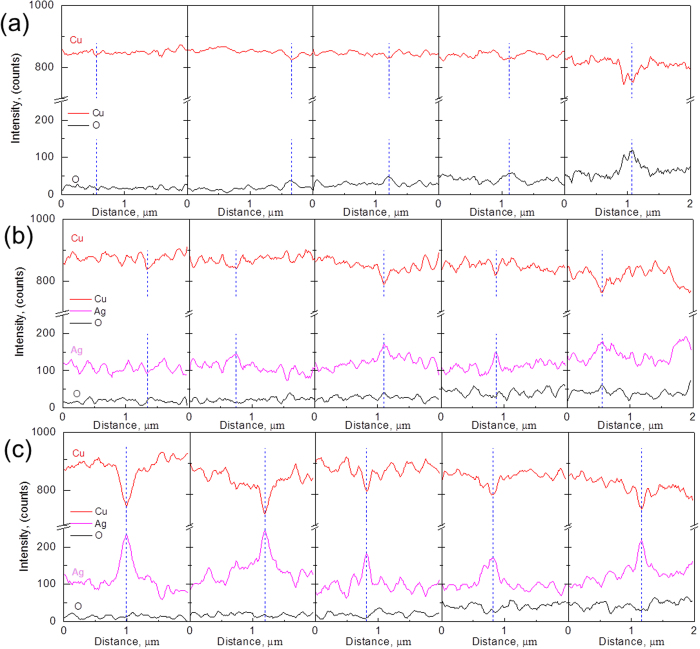
Line-scan analysis. (**a**–**c**) Line-scan profiles of the white lines (**a**) in Fig. 5(a–e), the crimson lines (**b**)in Fig. 6(a–e) and the dark blue lines (**c**) in Fig. 7(a–e).

Figure 10 shows EDS spectra across typical GBs of the three powders, where the positions of GB are highlighted by blue dashed lines. For comparison, the relative intensity of each element is plotted in the same range. It can be clearly observed that overall intensity of O for the pure Cu particle increases significantly in the progress of oxidation. This is particularly evident at GBs where O peak intensity (corresponding valley for Cu intensity) is observed obviously and becomes much more pronounced with the oxidation proceeding. In contrast, for the as-atomized Cu-Ag, O peaks in both GBs and the interior of matrix are not so protruding (Fig. 10a), suggesting that oxidation resistance is improved by adding Ag. Some Ag peaks are observed along GBs. However, O intensity is relatively higher at GBs compared to the matrix implying that oxidation at GBs is not suppressed completely. For the aging-treated powder, strong Ag peaks together with O valleys at GBs are clearly observed throughout oxidation, suggesting strong inhibition of oxidation at GBs by Ag precipitation. In addition, in grain matrix, O intensity is a little smaller for the aging-treated Cu-Ag particles than the as-atomized Cu-Ag ones.

## Discussion

It can be inferred from the aforementioned results that the addition of Ag into Cu and the subsequent aging treatment together contribute to high electrical conductivity and high oxidation resistance. This is attributable to the presence of the continuous Ag network along GBs, which inhibits preferential oxidation of GBs and then enhances overall oxidation resistance of powder without sacrificing electrical conductivity. The enhanced oxidation resistance can be ascribed to high dissociation pressure of Ag_2_O compared to Cu_2_O and CuO^27^,^28^. This means that the preferential oxidation of GB could be inhibited by coverage of Ag phase. On the contrary, pure Cu particles suffer from a rapid oxidation throughout the oxidation process because of the poor oxidation resistance of GBs relative to that of grain interiors. This agrees well with the above results shown in Fig. 5.

The relationship between the mass change and oxidation temperature/time in our case can be expressed by^29^:


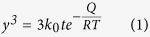


where *y* is mass change (Δ*m*/*m*_0_) at time *t*; *k*_0_ is the temperature-independent pre-exponential; *Q* is the energy of activation; *T* is the absolute temperature; and *R* is the universal gas constant. The dependence of oxidation rate on temperature can also be defined by the Arrhenius equation,


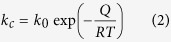


where *k*_c_ represents cubic rate constant (min^−1^) at each temperature for three powders. Substituting *k*_c_ into Eq. (1) yields a linear expression,


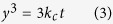


**Figure 11:**
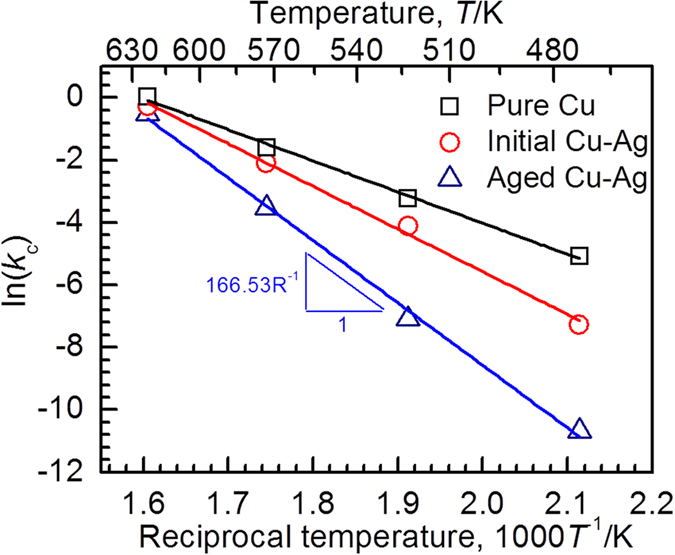
Arrhenius plot. Arrhenius plot of cubic rate constants during the oxidation process for the three different types of powders at air.

Thus, the oxidation rate can be determined by Eq. (3). Table 1 lists the *k*_c_ values for the linear segment of curves together with the correlation coefficient value, *r*^2^. All the *r*^2^ values approach 1, indicating that the cubic oxidation law is reasonably applicable to the oxidation behavior. By plotting *k*_c_ against reciprocal of temperature, the activation energy of oxidation can be generated from the slope of every fitting line, as shown in Fig. 11. The activation energies of the Cu and as-atomized Cu-Ag powders are calculated to be 83 and 114 kJ/mol, respectively. In contrast, the activation energy of the aged Cu-Ag powder is much higher (167 kJ/mol), implying that the aging-treated Cu-Ag powder undergoes a slow oxidation (Table 1).

The activation energies of oxidation, *Q* for the three powders are compared with those of bulk Cu—oxygen-free high-conductivity Cu (OFHC) (99.9999 pct (6 N) Cu and 99.99 pct (4 N) Cu), as listed in Table 2. A typical Q for the pure Cu has been reported to be in the range of 40–85 kJ/mol at 300–550 °C^30^,^31^,^32^, and 158–173 kJ/mol at 800–1050 °C^31^,^33^. Our present pure Cu powder oxidized at 200–350 °C also has a *Q* of 83 kJ/mol, which agrees with the previously reported value.

It is widely accepted that GB diffusion predominates during oxidation of Cu at low temperature (<550 °C), while at high temperature (>900 °C) lattice diffusion is dominated during oxidation^33^,^34^. It is interesting to find that the aging-treated Cu-Ag at 200–350 °C demonstrates a *Q* value as high as that of pure Cu at high temperature, implying that the oxidation of aged Cu-Ag powder is possibly linked to the lattice diffusion of Cu, since the diffusion of Cu at GBs is blocked due to the continuous distribution of Ag phase. The Q of as-atomized Cu-Ag powder at 200–350 °C is close to that of 6 N copper at intermediate temperatures of 600–850 °C, where the oxidation relies on both GB diffusion and lattice diffusion^33^,^34^.

The successful use of inert and conducting Ag rich in GBs of a Cu-Ag powder represents a remarkable step forward in realizing high electrical conductivity and high oxidation resistance for a broad range of electrical transfer applications. Our results demonstrate, for the first time, that the unique resultant microstructure leads to the effective inhibition of oxidation of the Cu powder while not sacrificing the electrical conductivity. Such an outstanding combination of oxidation resistance and electrical conductivity in Cu-Ag powders holds substantial promise as a new material platform for further development of electrical transfer powder.

## Methods

### Sample preparation

Two kinds of Cu-based powders, pure Cu (>99.8%) and Cu-5Ag (mass%) alloy, were applied as starting materials. The two powders were fabricated by a Hermiga gas atomizer (PSI, Hailsham, East Sussex, UK), which enabled us to obtain extremely low oxygen content (<0.0080 mass%, see in Table S1), good spherical shape (Fig. 1a,b) and comparable size distribution after sifting (Fig. S2). A graphite crucible containing ingots was placed into the atomizer, which was then heated to 1200 °C at a rate of 50 °C/min. High-purity argon gas (≥99.99%) emitted at a constant pressure of 2.0 MPa and high-vacuum reactor (1.7 Pa) were used to ensure the low oxygen content. The as-atomized Cu-Ag alloy powder (5 g at each condition) was contained in an alumina crucible and placed into a tubular resistance furnace, being aged with a constant hydrogen flow rate of 500 ml/min at 100 °C, 125 °C, 150 °C, 200 °C and 250 °C for the duration of 5, 10, 30, 60, 90 and 120 min, respectively. One end of the furnace was equipped with a H_2_ inlet and at the other end H_2_ was ignited in air. Before H_2_ flowing into the tubular furnace, N_2_ was flowed for 5 min to get rid of O_2_. To probe oxidation process of the powders, we conducted TGA analyses in dry air using a SDT Q600analyzer(TA Instruments, USA)under a heating rate of 30 °C/min from room temperature to 400 °C. The isothermal oxidation test was carried out at 200, 250, 300 and 350 °C for up to 3 h. The electrical conductivity of the powders was measured by using FT-300 powder conductivity tester (Rooko Instrument Co., China).

### Structural characterization

To characterize microstructure of the particles from the cross-section direction, the powders were first cold-mounted in epoxy, followed by grinding with 1000 and 2000-mesh SiC abrasive paper and polishing in OP-S solution for 30 min. Microstructural observation from both the cross section and surface of the particles was conducted using scanning electron microscopy (SEM; S-3400N, Hitachi, Tokyo, Japan) equipped with energy-dispersive spectroscopy (EDS) and an electron probe micro-analyzer (EPMA). The particle size of 10–30 μm was chosen for the observation.

## Additional Information

**How to cite this article**: Li, J. *et al*. Ultrahigh Oxidation Resistance and High Electrical Conductivity in Copper-Silver Powder. *Sci. Rep.* **6**, 39650; doi: 10.1038/srep39650 (2016).

**Publisher's note:** Springer Nature remains neutral with regard to jurisdictional claims in published maps and institutional affiliations.

## Supplementary Material

Supplementary Information

## Figures and Tables

**Table 1:** Cubic rate constant *k*_c_, correlation coefficient *R* ^2^, and activation energy *E*_a_ for the three types of powders.

Cubic constant *k*_c_ /min^−1^, (*R*^2^)	200 °C	250 °C	300 °C	350 °C	*E*_*a*_, kJ/mol
Pure Cu powder	6.23. × 10^−3^ (0.99698)	1.34 × 10^−2^ (0.99658)	6.85 × 10^−2^ (0.99849)	3.47 × 10^−1^ (0.99992)	83.03 (0.99619)
Initial Cu-Ag powder	6.90 × 10^−4^ (0.99939)	5.40 × 10^−3^ (0.99947)	4.07 × 10^−2^ (0.99969)	2.58 × 10^−1^ (0.99542)	113.72 (0.99504)
Aged Cu-Ag powder	2.29 × 10^−5^ (0.99515)	6.90 × 10^−4^ (0.99691)	2.95 × 10^−2^ (0.99735)	1.98 × 10^−1^ (0.99798)	166.53 (0.99667)

**Table 2:** Activation energies for the different Cu-related materials.

Starting material	Atmospheric condition (1 atm)	Temperature range (°C)	Activation energy (kJ/mol)	Ref.
OFHC (99.9%)	O_2_	300–550	84	Valensi^31^
		500–900	158	
99.90%	Air	250–500	71	O’reilly^32^
99.99%	O_2_	350–550	52	Zhu^33^
		600–900	95	
		900–1050	173	
100.00%	O_2_	350–550	40	Zhu^33^
		600–850	111	
		850–1050	173	
Pure Cu powder	Air	200–350	83	Present work
Initial Cu-Ag powder	Air	200–350	114	
Aged Cu-Ag powder	Air	200–350	167	
