# Supplementary material for: Ultrahigh Oxidation Resistance and High Electrical Conductivity in Copper-Silver Powder
Source: Sci Rep. 2016 Dec 22;6:39650. doi: 10.1038/srep39650 (PMC5177874; doi:10.1038/srep39650)

Ultrahigh Oxidation Resistance and High Electrical Conductivity in Copper-Silver Powder

Jiaxiang Li[1], Yunping Li[2, 1*], Zhongchang Wang[3], Huakang Bian[4], Yuhang Hou[4], Fenglin Wang, Guofu Xu[1], Bin Liu[2] & Yong Liu[2]

[1]*School of Materials Science and Engineering, Central South University, Changsha, China*

[2] *State Key Lab for Powder Metallurgy, Central South University, Changsha, China*

[3]*Advanced Institute for Materials Research, Tohoku University, 2-1-1 Katahira, Aoba-ku, Sendai 980-8577, Japan*

[4]*Institute for Materials Research, Tohoku University, 2-1-1 Katahira, Sendai, Miyagi 980-8577, Japan*

1 **Supplementary Figure S1.** Thermodynamic phase diagram for Ag_2O, CuO and Cu_2O.

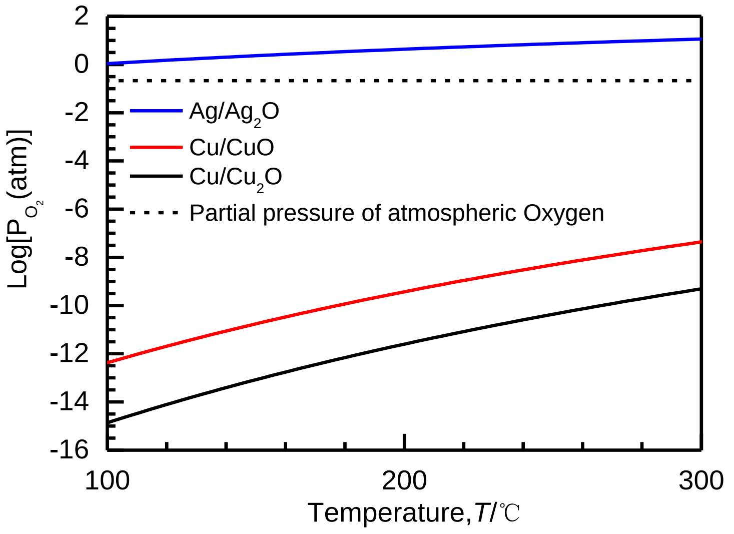

2 **Supplementary Table S1.** Composition of atomized pure Cu and Cu-Ag powder.

The wet chemical method with KI-$Na_2S_2O_3$ aqueous solutions, was employed to determine the Cu content in two powders, and Qualitative analyses for impurities were made by X-ray fluorescence spectrometer (XRF; Axios^{mAX} , PANalytical, Netherland). Ag content for Cu-Ag powder was measured using inductively coupled plasma optical emission spectrometer (ICP-OES, Spectro Blue Sop, German). Oxygen content was analyzed quantitatively on a nitrogen/oxygen/hydrogen determinator (TCH600, LECO Corporation, St. Joseph, Michigan, USA). All of these results are as tabulated in Table S1.

Powder composition (wt.%)	Cu	Ag	O	Impurities<br>(Al, Fe, S, Si)
Pure copper	99.70	N/A	0.0055	Bal.
Cu-Ag alloy powder	94.07	5.144	0.0072	Bal.

3 **Supplementary Figure S2.** Particle size distribution of atomized pure Cu and Cu-Ag powder.

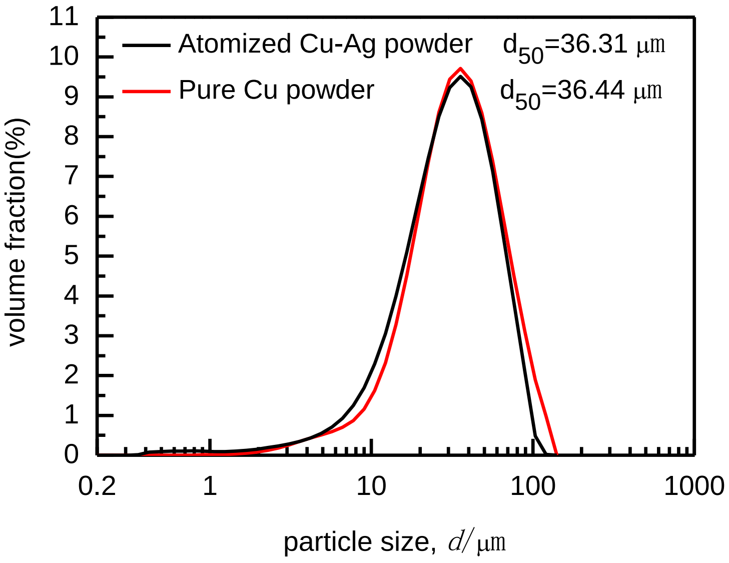

Supplement: Supplementary Information [file srep39650-s1.pdf]
